# Impact of creatinine production on the agreement between glomerular filtration rate estimates using cystatin C-derived, and 4- and 6-variable Modification of Diet in Renal Disease (MDRD), and Chronic Kidney Disease Epidemiology Collaboration (CKD-EPI) equations

**DOI:** 10.3109/03009734.2012.696154

**Published:** 2012-10-30

**Authors:** Esperanza F. Hermida-Cadahia, Natalia Lampon, J. Carlos Tutor

**Affiliations:** Unidad Monitorización Fármacos, Laboratorio Central, Hospital Clínico Universitario, Instituto de Investigación Sanitaria (IDIS), 15706 Santiago de Compostela, Spain

**Keywords:** CKD-EPI, cystatin C, glomerular filtration rate, impaired creatinine production, MDRD

## Abstract

**Background.:**

It has recently been reported that patient selection has a strong impact on the agreement between glomerular filtration rate (GFR) estimates from serum cystatin C and creatinine. The aim of our study was to evaluate the effect of creatinine production rate (CPR) on this subject.

**Material and methods.:**

GFR was estimated from serum cystatin C and from creatinine using the 4- and 6-variable Modification of Diet in Renal Disease (MDRD), and Chronic Kidney Disease Epidemiology Collaboration (CKD-EPI) equations in 50 healthy subjects, 43 patients with renal failure, 794 kidney and 104 liver transplant recipients, 61 patients with heart failure, 59 patients with biliary obstruction, and 113 critically ill patients.

**Results.:**

In the 295 patients with impaired CPR (< 900 mg/24 h/1.73 m^2^), discordances of more than 40% between GFR_MDRD4_ and GFR_cystatinC_ were observed in 38% of cases, between GFR_MDRD6_ and GFR_cystatinC_ in 22%, and between GFR_CKD-EPI_ and GFR_cystatinC_ in 27% (in all cases due to GFR overestimation from creatinine). In the 929 patients with maintained CPR (> 900 mg/24 h/1.73 m^2^), greater discordances than 40% between GFR_MDRD4_ and GFR_cystatinC_ were observed in 8% of cases, between GFR_MDRD6_ and GFR_cystatinC_ in 9%, and between GFR_CKD-EPI_ and GFR_cystatinC_ in 7% (in the major part of cases due to GFR overestimation from cystatin C).

**Conclusion.:**

The main source of differences of more than 40% between GFR estimates from serum creatinine and cystatin C is a GFR overestimation in patients with low CPR and GFR underestimation in patients with high CPR by the creatinine-derived equations.

## Introduction

The estimation of glomerular filtration rate (GFR) based on both serum cystatin C and creatinine is considered preferable to GFR prediction based on either cystatin C or creatinine (1–3); however, this may not be the case in several clinical contexts (3). The diagnostic performance of estimated GFR from serum creatinine is reduced in patients with an abnormally low or high muscle mass, malnourishment, or liver disease. In these conditions the performance of GFR estimation from cystatin C is generally unaltered; however, the performance of estimated GFR from this biochemical variable may be decreased in patients with thyroid dysfunction or treated with large doses of glucocorticoids (3,4). Similarly, the half-life of serum cystatin C will be three times shorter than creatinine, leading to an earlier attainment of a new steady state (5). Consequently, cystatin C has been proposed as a good real-time GFR marker in unstable critically ill patients (6,7), reflecting acute GFR changes more rapidly than creatinine (8–10). According to the suggestions of Grubb et al. (3), if the difference between the GFR values predicted from creatinine and cystatin C is 40% or more, the combined use of both markers has an unacceptable diagnostic performance. Possible conditions invalidating either creatinine or cystatin C should be evaluated, and GFR may be best estimated only on the non-invalidated marker (3).

Larsson et al. (11) found that selection of patients has a strong impact on the agreement between estimated GFR from serum cystatin C and creatinine, with better concordance in primary care and cardiology patients than in patients from oncology wards and neurosurgical intensive care, in whom the use of creatinine led to a significant GFR overestimation. Concordant results were described for a population of patients treated in a general intensive care unit, with an increase of the number of cases identified as renal insufficiency when GFR estimation was made from serum cystatin C (12). The estimation of GFR combining creatinine- and cystatin C-based results in patients with critical illness as suggested by Chao (13) is probably inaccurate because, as emphasized by Larsson (14), the differences between GFR estimates from cystatin C and creatinine may be profound, and we do not know which of the two markers is correct. However, serum cystatin C, which is not significantly influenced by systemic inflammation (15), anthropometric data, or muscle variation (3,4), is generally considered to be a better GFR marker than creatinine in critically ill patients (6,7,16–19).

In accordance with previous results (20), creatinine production impairment may be responsible for significant creatinine GFR overestimation with respect to values predicted from cystatin C. The aim of our study was to investigate, in patients with different pathophysiological conditions, the impact of creatinine production on the agreement between GFR estimates from serum cystatin C and creatinine using the 4- and 6-variable Modification of Diet in Renal Disease (MDRD) and Chronic Kidney Disease Epidemiology Collaboration (CKD-EPI) equations.

## Patients and methods

A total of 1224 serum samples were analysed from 50 healthy subjects, 43 patients with renal failure, 794 kidney and 104 liver transplant recipients, 61 patients with heart failure, 59 patients with biliary obstruction, and 113 ill patients hospitalized in a general intensive care unit. This study was carried out according to the good practice rules for investigation in humans of the Conselleria de Sanidade (Regional Ministry of Health) of the Xunta de Galicia, Spain.

Serum cystatin C was determined by particle-enhanced nephelometric immunoassay (PENIA) using the N Latex Cystatin C reagent in a BN ProSpec® nephelometer (Siemens Health Care Diagnostics, Inc.), and estimation of GFR from serum cystatin C concentrations was carried out using the equation of Hoek et al. (21). Creatinine was determined by a kinetic alkaline picrate method in an Advia 2400 Chemistry System (Siemens Health Care Diagnostics, Inc.). The same analyser was used to determine serum albumin and urea concentrations. GFR estimation from serum creatinine was made using the 4-variable (age, sex, race, and serum creatinine) MDRD equation (22), the 6-variable (age, sex, race, and serum creatinine, urea, and albumin) MDRD equation (23), and the CKD-EPI equation (24). The creatinine production rate (CPR) was calculated assuming that essentially all creatinine produced in the body is eliminated via the kidneys using the expression: CPR (mg/24 h/1.73 m^2^) = GFR × serum creatinine concentration, where GFR corresponds to estimated values from cystatin C (25,26).

Statistical analysis was performed using the StatGraphics Plus (v. 5.0) program. The Shapiro–Wilks test was applied to check for normality, and the Pearson correlation coefficient was used when the data had Gaussian distribution; otherwise, the Spearman correlation coefficient was used. Results were expressed as mean ± SEM (median). The regression study was carried out using the method of Passing–Bablock, and dispersion of data was evaluated by means of the standard error of estimate (Sy.x). The results were also compared using the Eksborg difference plots (27).

## Results

**Figure 1.**
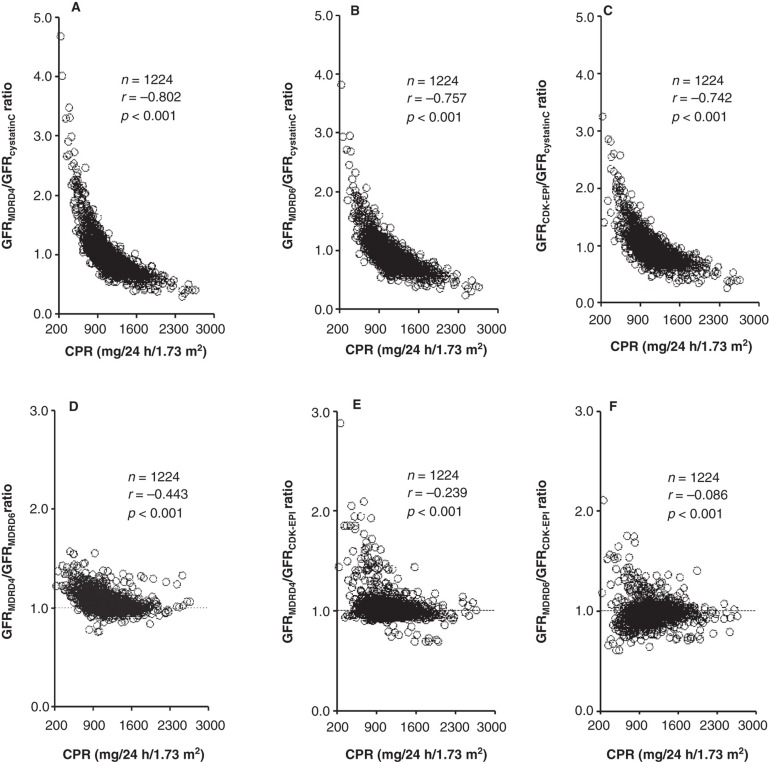
Relationship of the ratios between the GFR estimates produced by the different cystatin C- and creatinine-derived equations with the creatinine production rate (CPR).

Figure 1A-F shows the association between CPR and the ratios of estimated GFR values using the creatinine-derived 4-variable MDRD (GFR_MDRD4_), 6-variable MDRD (GFR_MDRD6_), and CKD-EPI (GFR_CKD-EPI_) equations, and cystatin C-derived (GFR_cystatinC_) equation for the total of cases studied. Except for the GFR_MDRD6_/GFR_CKD-EPI_ ratio (Figure 1F), significant negative correlations with CPR were obtained.

Table I shows the results obtained for the CPR and estimated GFR from serum creatinine and cystatin C in the different groups of healthy subjects and patients. All of the patient groups have significantly lower CPR than the control group (*P* < 0.001), and the group of critical care patients has a significantly lower CPR than the other groups of patients (*P* < 0.001). Significant correlations were obtained for the CPR with serum albumin concentration (*r* = 0.387, *P* < 0.001) and blood urea nitrogen (BUN)/creatinine ratio (*r* = –0.405, *P* < 0.001).

**Table I.** Creatinine production rate (CPR) and glomerular filtration rate (GFR) estimates from serum cystatin C and creatinine in the groups of controls (C), patients with renal failure (RF), renal transplant (RTx), heart failure (HF), biliary obstruction (BO), liver transplant (LTx), and critical illness (CI).

	*n*	Age (years)	CPR (mg/24 h/1.73 m^2^)	Albumin (g/L)	GFR_MDRD4_ (mL/24 h/1.73 m^2^)	GFR_MDRD6_ (mL/24 h/1.73 m^2^)	GFR_CKD-EPI_ (mL/24 h/1.73 m^2^)	GFR_cystatinC_ (mL/24 h/1.73 m^2^)
C	50	53.4 ± 2.8 53	1596.0 ± 317.3 (1604.5)	45.2 ± 0.4 (45.0)	76.2 ± 13.8 (77.5)	74.9 ± 13.5 (72.4)	84.5 ± 15.1 (85.9)	115.7 ± 20.6 (113.2)
RF	43	65.6 ± 2.6 70	1201.0 ± 322.7 (1206.6)	37.7 ± 0.7 (37.0)	10.0 ± 5.2 (8.3)	9.7 ± 4.7 (8.7)	9.0 ± 4.8 (7.7)	14.1 ± 5.9 (12.6)
RTx	794	47.0 ± 2.0 41	1166.7 ± 318.4 (1123.6)	43.6 ± 0.1 (44.0)	53.4 ± 20.9 (52.0)	51.8 ± 20.5 (50.6)	53.8 ± 21.1 (52.4)	59.7 ± 24.3 (57.5)
HF	61	74.3 ± 1.8 78	1063.3 ± 73.5 (1082.4)	36.0 ± 0.7 (36.0)	53.5 ± 23.8 (53.0)	51.5 ± 22.9 (49.7)	52.6 ± 23.0 (53.0)	65.7 ± 27.2 (62.6)
BO	59	63.6 ± 2.9 65	1075.7 ± 368.8 (1020.3)	30.5 ± 0.7 (30.0)	77.6 ± 31.5 (74.1)	71.3 ± 29.0 (72.7)	73.4 ± 27.0 (71.8)	76.4 ± 24.3 (77.2)
LTx	104	52.5 ± 1.7 55	949.4 ± 293.1 (943.2)	34.1 ± 0.7 (34.0)	73.8 ± 42.4 (64.2)	64.9 ± 33.7 (58.8)	69.3 ± 24.9 (63.6)	60.0 ± 24.0 (59.5)
CI	113	61.6 ± 2.1 59	853.8 ± 340.5 (798.1)	31.3 ± 0.5 (31.0)	131.9 ± 66.0 (127.8)	111.5 ± 56.1 (109.2)	96.9 ± 32.6 (99.7)	91.9 ± 40.7 (93.2)

For a better characterization of the patients with significantly impaired creatinine production, a cut-off value was considered for the CPR (900 mg/24 h/1.73 m^2^) corresponding to 1.0 percentile (instead of the usual 2.5 percentile) of the results obtained in the group of 50 healthy subjects included in our study. The prevalence of CPR < 900 mg/24 h/1.73 m^2^ in the different groups of patients was 3% in heart failure, 19% in kidney transplant, 23% in renal failure, 31% in biliary obstruction, 46% in liver transplant, and 65% in critical illness. In relation to the patients with CPR > 900 mg/24 h/1.73 m^2^ (*n* = 929), the patients with CPR < 900 mg/24 h/1.73 m^2^ (*n* = 295) present analogous age (58.3 ± 0.9 (59.0) years versus 56.2 ± 0.5 (56.7) years), but significantly lower albumin concentration (36.6 ± 0.4 (38.0) g/L versus 41.7 ± 0.2 (43.0) g/L, *P* < 0.001), and higher BUN/creatinine ratio (28.9 ± 0.7 (26.6) versus 21.7 ± 0.3 (20.7), *P* < 0.001).

**Figure 2.**
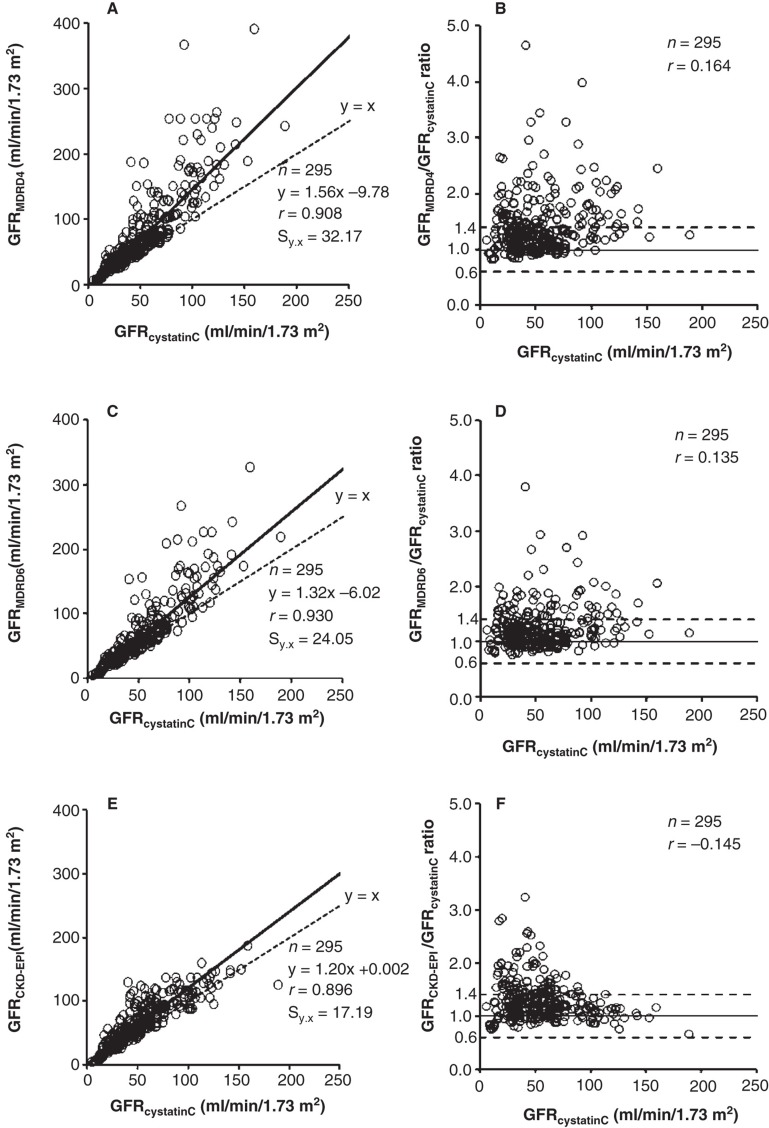
Correlation and linear regression (A, C, E) and Eksborg difference plots (B, D, F) between the GFR estimates from cystatin C and creatinine in patients with impaired creatinine production (CPR < 900 mg/24 h/1.73 m^2^). The dotted lines in the difference plots correspond to the proposed difference limits (≤ 40%) between GFR estimates from cystatin C and creatinine for its combined use as arithmetic mean (3).

Figure 2 (A, C, E) shows regression lines and corresponding standard error of estimates between the estimated GFR values from cystatin C and creatinine in patients with impaired creatinine production (CPR < 900 mg/24 h/1.73 m^2^). As indicated in the correspondent difference plots (B, D, F), differences of more than 40% between GFR_MDRD4_ and GFR_cystatinC_ were observed in 38% of cases, between GFR_MDRD6_ and GFR_cystatinC_ in 22%, and between GFR_CKD-EPI_ and GFR_cystatinC_ in 27% (in all cases due to GFR overestimation from creatinine).

**Figure 3.**
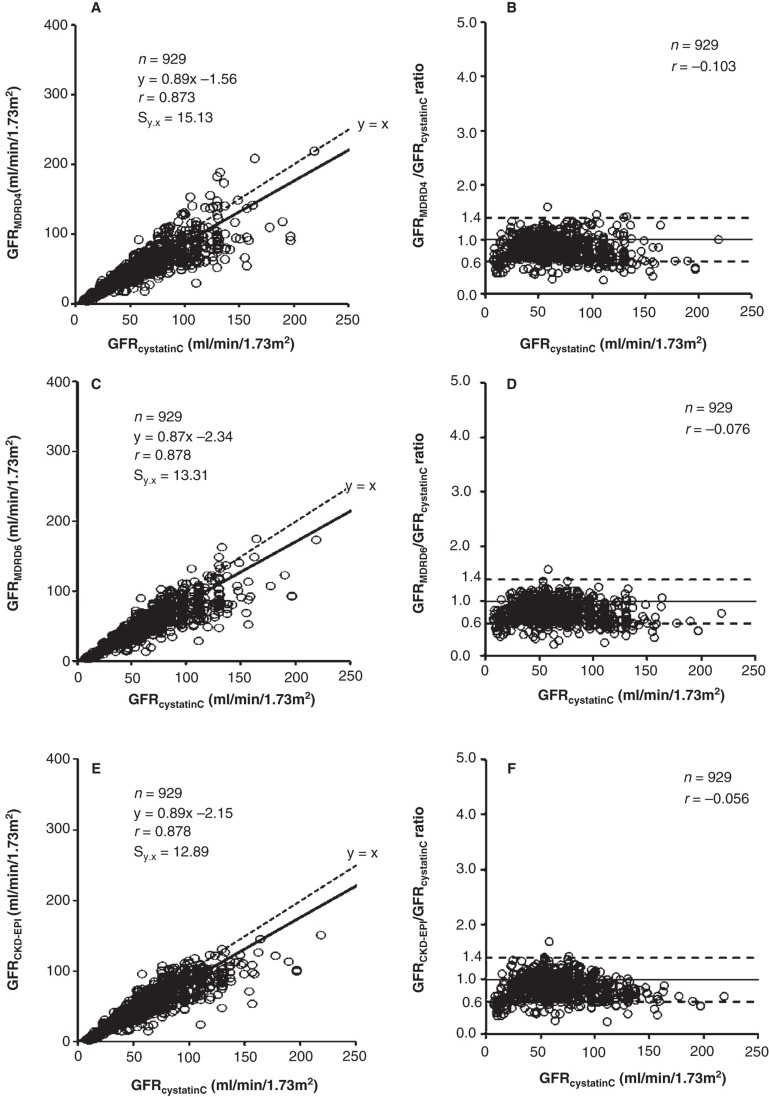
Correlation and linear regression (A, C, E) and Eksborg difference plots (B, D, F) between the GFR estimates from cystatin C and creatinine in patients with maintained creatinine production (CPR > 900 mg/24 h/1.73 m^2^). The dotted lines in the difference plots correspond to the proposed difference limits (≤ 40%) between GFR estimates from cystatin C and creatinine for its combined use as arithmetic mean (3).

In the patients with preserved creatinine production (CPR > 900 mg/24 h/1.73 m^2^), lower slopes, intercepts, and standard error of estimates values were obtained for the regression lines (Figure 3 A, C, E). Similarly, as indicated in the difference plots (B, D, F), differences of more than 40% between GFR_MDRD4_ and GFR_cystatinC_ were only observed in 8% of cases, between GFR_MDRD6_ and GFR_cystatinC_ in 9%, and between GFR_CKD-EPI_ and GFR_cystatinC_ in 7% (in the major part of cases due to GFR overestimation from cystatin C).

## Discussion

Creatine is metabolized to creatinine by a non-enzymatic cyclization especially in skeletal muscle, and the serum creatinine concentration is a function of its production and renal excretion. Consequently, decreased CPR from loss of muscle mass, malnourishment, or diminished hepatic creatine formation in liver disease explains that in these cases serum creatinine may be a poor GFR predictor (3,4,25,26). However, cystatin C may be a suitable marker for GFR estimation in these clinical conditions (3,4,28,29).

In all likelihood, 24-hour urinary creatinine excretion is an accurate reflection of muscle mass and creatinine generation (30,31). As the measured daily urinary creatinine excretion was not available in our patients, CPR was calculated as previously described (25,26), estimating GFR from cystatin C by means of the equation of Hoek et al. (21). Although this equation was developed in 2003 (21), and calibration of the PENIA cystatin C assay may have changed over time (32), the Hoek formula has recently been favourably evaluated by different authors (33–36).

Highly significant negative correlations of the ratios between estimated creatinine- and cystatin C-based GFR values with the CPR were obtained (Figure 1 A, B, and C). Likewise, data included in Table I show that in the different groups of controls and patients considered, the mean (median) overestimation of GFR using creatinine-derived equations (mainly 4-variable MDRD) with respect to the cystatin C-derived equation increases with decrease of CPR and albumin levels (mainly ill patients group), and GFR underestimation increases in parallel with CPR and albumin levels (mainly control group). Figures 2 and 3 show significant differences obtained for the regression and dispersion between the GFR estimates from serum cystatin C and creatinine in cases with impaired or maintained CPR. In patients with CPR < 900 mL/min/1.73 m^2^, a difference of more than 40% between creatinine- and cystatin C-derived GFR estimates was in all cases due to an overestimation of GFR values from creatinine (Figure 2 B, D, F); however, in patients with CPR > 900 mL/min/1.73 m^2^, a difference of more than 40% was due in the majority of cases to an underestimation of GFR values from creatinine (Figure 3 B, D, F). In patients with maintained creatinine production, the concordances between estimated GFR from cystatin C and 4-and 6-variable MDRD and CKD-EPI equations were analogous; nevertheless, in cases with impaired creatinine production, a poorer concordance was obtained for 4-variable MDRD. These results are consistent with the assumption that creatinine production is an important modulator of the accuracy of creatinine-derived equations, which overestimate GFR in patients with low creatinine production and underestimate GFR in patients with high creatinine production (30).

GFR estimation is a serious problem in patients with critical illness, as mortality is increased dramatically when complicated with acute kidney injury. As reported by Larsson et al. (11) differences in patient selections have a strong impact on the agreement between cystatin C and 4-variable MDRD estimated GFR. Specifically, in patients treated in a general intensive care unit, the use of cystatin C instead of creatinine will significantly increase the proportion of patients identified with decreased GFR from 46% to 92% (12). For the 113 critical care patients included in our study, higher correlation coefficients were obtained with respect to the total patients group between the CPR and GFR_MDRD4_/GFR_cystatinC_ (*r* = –0.875, *P* < 0.001), GFR_MDRD6_/GFR_cystatinC_ (*r* = –0.817, *P* < 0.001), and GFR_CKD-EPI_/GFR_cystatinC_ (*r* = –0.807, *P* < 0.001) ratios. Patients with critical illness have a progressive decline of creatinine production as a result of malnutrition, greater co-morbidity, sepsis, and loss of muscle mass worsened by subclinical hepatic injury (30,37,38). Consequently, low CPR is highly prevalent in intensive care unit patients (65% of our patients according to the data indicated above), and this fact may explain the results of Larsson et al. (11,12).

In the major part of cases with maintained creatinine production, the differences between creatinine- and cystatin C-derived GFR estimates were lower than 40%, permitting an improved estimation of GFR through the combined use (arithmetic mean) of cystatin C and creatinine estimates in accordance with the recommendations of Grubb et al. (3); however, in cases with impaired creatinine production, GFR overestimation from creatinine with respect to cystatin C is frequently greater than 40% (6-variable MDRD and CKD-EPI has moderately better concordance than 4-variable MDRD), and one of the two markers should be invalidated (probably creatinine if thyroid dysfunction and glucocorticoids administration may be excluded). In any case, if the source of a large discordance between cystatin C-and creatinine-derived values is not identified, both GFR estimates may provide an unacceptable performance, and an invasive gold standard measurement of GFR might be required.

Poggio et al. (39) have reported that GFR estimates from serum creatinine using the 4-variable MDRD equation are not reliable measurements in ill hospitalized patients, especially those with a BUN/creatinine ratio greater than 20, with the 6-variable MDRD equation offering moderately improved performance. Elevated BUN/creatinine ratio is caused more often by an increase of urea generation than a decrease of serum creatinine; however, these authors assume that in ill patients an increased BUN/creatinine ratio could be caused by decreased creatinine production (39). In the 1224 patients studied, the group of cases with impaired CPR has a greater BUN/creatinine ratio than the group with preserved CPR (*P* < 0.001). Although a significant negative correlation was obtained between both biochemical variables (*r* = –0.405, *P* < 0.001), the correspondent determination coefficient (*r^2^* = 0.164) indicates that differences in CPR only would explain 16% of the interindividual variability of the BUN/creatinine ratio. In accordance with this fact, poor correlation coefficients were obtained between the BUN/creatinine and GFR_MDRD4_/GFR_cystatinC_ (*r* = 0.305, *P* < 0.001), GFR_MDRD6_/GFR_cystatinC_ (*r* = 0.139, *P* < 0.001), and GFR_CKD-EPI_/GFR_cystatinC_ (*r* = 0.249, *P* < 0.001) ratios. In the 113 critical care patients included in our study, the coefficient of determination obtained between the BUN/creatinine ratio and CPR (*r^2^* = 0.141) indicates a modest degree of association between these variables, even in groups of critically ill patients with a high prevalence of decreased creatinine production. Consequently, the BUN/creatinine ratio does not appear to be a useful marker of impaired CPR, as well as a reliable predictor of unacceptable discordances between estimated GFR values from serum creatinine and cystatin C (data not shown).

In conclusion, according to our results, the main source of unacceptable differences of more than 40% between GFR estimates from serum creatinine and cystatin C (3) is an unusual CPR. Calculation of two GFR estimates based on each of both biochemical variables may be preferable to the use of a combined equation resulting in a single estimate. Evaluation of the discrepancy between the two GFR estimates can help to choose the more appropriate value: arithmetic mean of estimated GFRs, creatinine-derived GFR estimates in patients with thyroid dysfunction or treated with glucocorticoids, or cystatin C-derived GFR estimates in patients with altered CPR. In cases with unexplained large discrepancies between GFR estimates from creatinine and cystatin C, the GFR determination using a gold standard method may be desirable.

To the best of our knowledge, there are no available data on the comparative use of gold standard GFR measurements in critical care patients, and further studies on this subject are necessary for a more accurate evaluation of the performance of estimated GFR from cystatin C in these patients.
